# FHODs: Nuclear tethered formins for nuclear mechanotransduction

**DOI:** 10.3389/fcell.2023.1160219

**Published:** 2023-05-04

**Authors:** Susumu Antoku, Thomas U. Schwartz, Gregg G. Gundersen

**Affiliations:** ^1^ Department of Pathology and Cell Biology, Columbia University, New York, NY, United States; ^2^ Department of Biology, Massachusetts Institute of Technology, Cambridge, MA, United States

**Keywords:** nucleus, LINC complex, FHOD, formins, mechanotransduction, actin filaments, nesprins

## Abstract

In this review, we discuss FHOD formins with a focus on recent studies that reveal a new role for them as critical links for nuclear mechanotransduction. The FHOD family in vertebrates comprises two structurally related proteins, FHOD1 and *FHOD3*. Their similar biochemical properties suggest overlapping and redundant functions. *FHOD1* is widely expressed, *FHOD3* less so, with highest expression in skeletal (*FHOD1*) and cardiac (*FHOD3*) muscle where specific splice isoforms are expressed. Unlike other formins, FHODs have strong F-actin bundling activity and relatively weak actin polymerization activity. These activities are regulated by phosphorylation by ROCK and Src kinases; bundling is additionally regulated by ERK1/2 kinases. FHODs are unique among formins in their association with the nuclear envelope through direct, high affinity binding to the outer nuclear membrane proteins nesprin-1G and nesprin-2G. Recent crystallographic structures reveal an interaction between a conserved motif in one of the spectrin repeats (SRs) of nesprin-1G/2G and a site adjacent to the regulatory domain in the amino terminus of FHODs. Nesprins are components of the LINC (linker of nucleoskeleton and cytoskeleton) complex that spans both nuclear membranes and mediates bidirectional transmission of mechanical forces between the nucleus and the cytoskeleton. FHODs interact near the actin-binding calponin homology (CH) domains of nesprin-1G/2G enabling a branched connection to actin filaments that presumably strengthens the interaction. At the cellular level, the tethering of FHODs to the outer nuclear membrane mechanically couples perinuclear actin arrays to the nucleus to move and position it in fibroblasts, cardiomyocytes, and potentially other cells. FHODs also function in adhesion maturation during cell migration and in the generation of sarcomeres, activities distant from the nucleus but that are still influenced by it. Human genetic studies have identified multiple *FHOD3* variants linked to dilated and hypertrophic cardiomyopathies, with many mutations mapping to “hot spots” in *FHOD3* domains. We discuss how FHOD1/3’s role in reinforcing the LINC complex and connecting to perinuclear actin contributes to functions of mechanically active tissues such as striated muscle.

## Introduction

Formins are family of cytoskeletal regulators that affect both actin and microtubules. In humans 15 different genes encode formins comprising seven different classes (Chesarone et al., 2010; Valencia and Quinlan, 2021). Many formins are regulated by Rho GTPases that bind to the formin and release it from an autoinhibitory state. This stimulates the cytoskeletal activities of the formins and targets them to sites in cellular membranes.

The FHOD subfamily of formins comprises two members in humans (FHOD1 and FHOD3) and single members in *D.* *melanogaster* and *C. elegans.* FHODs do not exhibit potent actin polymerizing activity similar to diaphanous formins, but instead are potent actin bundlers (Bechtold et al., 2014). Also distinguishing FHODs from other formins is their mechanism of activation, which involves phosphorylation rather than Rho GTPase binding. These data have pointed to a unique mode of action of FHOD formins. Here, we discuss recent structural, biochemical and cell biological evidence revealing that FHOD formins are tethered to the nucleus by interacting directly with nesprins of the LINC complex in the outer nuclear membrane. We consider how FHODs contribute to the regulation of nuclear mechanics and mechanotransduction and how disruptions of FHOD either by mutations in genes encoding FHODs or changes in expression disrupt these functions and cause disease.

### FHOD1/3 discovery, expression, and structure

Formin homology 2 domain-containing protein 1 (FHOD1), initially named formin homolog overexpressed in spleen (FHOS1), was first discovered as an interactor of the transcription factor AML-1B from yeast two-hybrid screening (Westendorf et al., 1999). *FHOD3* (initially named FHOS2) was identified based on high protein sequence homology between its formin homology 2 (FH2) domain and other regions to those in FHOD1 (Kanaya et al., 2005). The protein sequence identity of FH2 domains in FHOD1 and *FHOD3* is 60% whereas that of FHOD1 and DIA1 formin is only 23%. This sequence similarity along with their distinct biochemical capabilities (see below) justifies grouping FHOD1/3 as a distinct formin subclass.

FHOD1 protein is expressed ubiquitously, but highest in lung, spleen, and skeletal muscle (Westendorf et al., 1999; Tojo et al., 2003; Sanematsu et al., 2019). A splice variant of FHOD1 containing an additional exon is expressed specifically in skeletal muscle (Tojo et al., 2003). *FHOD3* protein is not as ubiquitous as FHOD1 (Kanaya et al., 2005), and is most abundant in heart where a specific slice form is expressed (Kanaya et al., 2005; Iskratsch et al., 2010; Kan et al., 2012; Antoku et al., 2019). Among the 15 human formins, *FHOD1* is most abundantly expressed and *FHOD3* the least as judged by mRNA levels in tissues (Krainer et al., 2013).

**FIGURE 1:**
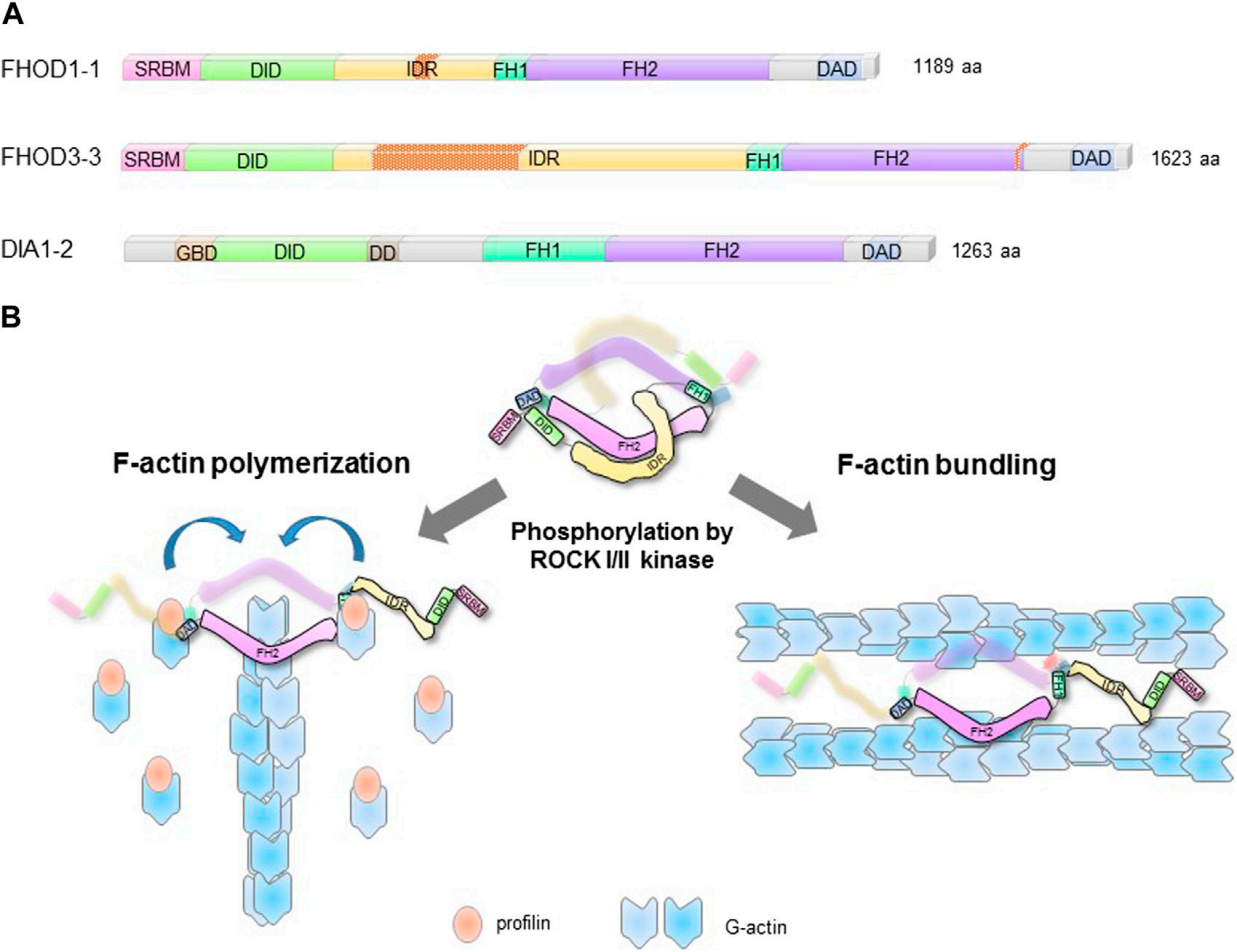
Structural domains and regulation of FHODs. **(A)**. Structural domains of striated-muscle splice forms of human FHOD1 (FHOD1-1), *FHOD3*, (*FHOD3*), and ubiquitously expressed DIA1 (DIA1-2). Nesprin-1/2 spectrin repeats binding module (SRBM); DAD interaction domain (DID); intrinsically disordered region (IDR); formin homology domains 1 and 2 (FH1/2); diaphanous autoregulatory domain (DAD); GTPase binding domain (GBD); dimerization domain (DD). The splice inserts in FHOD1-1 and *FHOD3*-3 are indicated by red stippling. **(B)**. FHOD F-actin reorganizing activities are autoinhibited by intramolecular interaction between DID and DAD. ROCKI/II phosphorylation of the DAD disrupts the autoinhibition and unleashes actin polymerization and F-actin bundling activities.

FHODs have the signature domains of diaphanous related formins (DRFs) including, formin homology domains 1 (FH1), 2 (FH2), and 3 (FH3), and the diaphanous autoregulatory domain (DAD) (Figure 1A). However, they lack the GTPase binding domain (GBD) of DRFs and are therefore distinct. The FH1 domain comprises three polyproline clusters that bind to profilin:G-actin complexes. It is shorter than the FH1 domain in DIA formins, but similar to those in other formins (Kovar et al., 2006). The FH1 domain supplies profilin:G-actin to the FH2 domain for polymerization (Figure 1B).

Formin FH2 domains typically interact with the barbed end of actin filaments and stimulate actin polymerization (Goode and Eck, 2007). Formin FH2 domains form dimers and this is critical for their ability to bind actin barbed ends and stimulate polymerization (Copeland et al., 2004; Moseley et al., 2004; Bartolini et al., 2008). Dimerization of the FH2 domain occurs by interaction between the so called “lasso” of one FH2 domain and the “post” of the other resulting in a toroid shaped, flexible dimer that is thought to toggle on the barbed end of the actin filament (Shimada et al., 2004; Xu et al., 2004; Otomo et al., 2005). Whether FHODs are dimeric is unknown, but based on conserved residues in the lasso and post regions and by their appearance as toroids in negative stain EM, they are predicted to be dimeric (Schonichen et al., 2013).

In many formins including FHODs, the DAD near the carboxy-terminus interacts intramolecularly with the FH3 (also referred to as the DAD interaction domain or DID) near the amino-terminus (Schonichen et al., 2006). This interaction suppresses F-actin polymerization activity of formins including FHODs (Alberts, 2001; Iskratsch et al., 2013; Patel et al., 2018; Antoku et al., 2019). For FHOD1/3, the DAD-DID interaction also suppresses F-actin bundling activity (Antoku et al., 2019).

FHOD formins have two unique elements compared to other formins. One of them is a spectrin repeat binding module (SRBM) in place of the GBD found in DRFs (Antoku et al., 2019; Lim et al., 2021). This module binds to specific SRs in the outer nuclear membrane proteins nesprin-1G and -2G (see below) (Kutscheidt et al., 2014; Antoku et al., 2019; Lim et al., 2021) The second is a predicted intrinsically disordered region (IDR) between the FH3/DID and the FH1 domain (Figure 1A). This region binds F-actin weakly (Takeya and Sumimoto, 2003; Schonichen et al., 2006) and is involved in regulation of F-actin bundling activity (Antoku et al., 2019). The IDR is the site of alternative splicing inserts in skeletal and cardiac muscle isoforms of FHOD1 and *FHOD3*, respectively, suggesting striated-muscle specific functions. Indeed, the cardiac splice isoform of *FHOD3* contains a 175-residue insert that binds to cardiac myosin binding protein C (Kanaya et al., 2005; Matsuyama et al., 2018). This interaction is necessary to localize cardiac *FHOD3* to the central zone of the myosin-containing A-band in cardiac sarcomeres.

#### FHOD formins actin polymerization activity

FHOD formins have not been shown to stimulate rapid actin polymerization like DRFs. For example, FHOD1 stimulates actin polymerization at less than 5% the rate of mDia1 (Patel et al., 2018; Antoku et al., 2019). Despite their weak stimulation of actin polymerization, mechanistic aspects of FHODs’ polymerization activity resemble those of other formins. It is dependent on profilin:G-actin complexes just like that of other formins (Paul and Pollard, 2008). FHOD1 blocks actin filament depolymerization upon dilution of actin monomers indicating that it binds the barbed end of actin filaments like other formins (Taniguchi et al., 2009; Schonichen et al., 2013). FHODs also possess conserved residues in the FH2 domain (e.g., I705 in human FHOD1 corresponding to I845 in mouse Dia1) that are required for actin polymerization (Shimada et al., 2004; Bartolini et al., 2008; Antoku et al., 2019).

The failure to detect robust actin polymerization with FHOD formins *in vitro* may reflect different intrinsic actin polymerization activities, or alternatively, that their actin polymerization activity is more tightly controlled. For example, mechanical pulling force may be required to enhance FHODs’ actin polymerization. Recent studies of mDia1 and yeast Bni1 have revealed a stimulatory effect on actin polymerization when a pulling force is applied to tethered versions of these formins (Jegou et al., 2013; Yu et al., 2017). As FHODs are tethered to the nucleus (see below), an applied force may enhance its actin polymerization activity. There may be other formins that show a latent polymerization activity. For example, Cdc12p in fission yeast shows strong barbed end capping activity but weak stimulation of actin polymerization (Kovar et al., 2003).

#### FHOD formins F-actin bundling activity

In contrast to their relatively weak actin polymerization activity, FHODs exhibit potent F-actin bundling activity *in vitro* and *in vivo* (Schonichen et al., 2013; Patel et al., 2018; Antoku et al., 2019). The bundling activity resides in the FH2 domain which is sufficient to bundle F-actin *in vitro* (Patel et al., 2018). The bundling activity of FHODs is strongly regulated by autoinhibition *via* the DAD domain (Antoku et al., 2019). Reflecting their biochemical activities, activated versions of FHODs lacking the DAD strongly bundle actin filaments in cells and unlike other formins, also localize to them. The localization to actin bundles in cells requires the IDR that precedes the FH1 domain (Figure 1A) (Takeya and Sumimoto, 2003; Schonichen et al., 2013).

**FIGURE 2:**
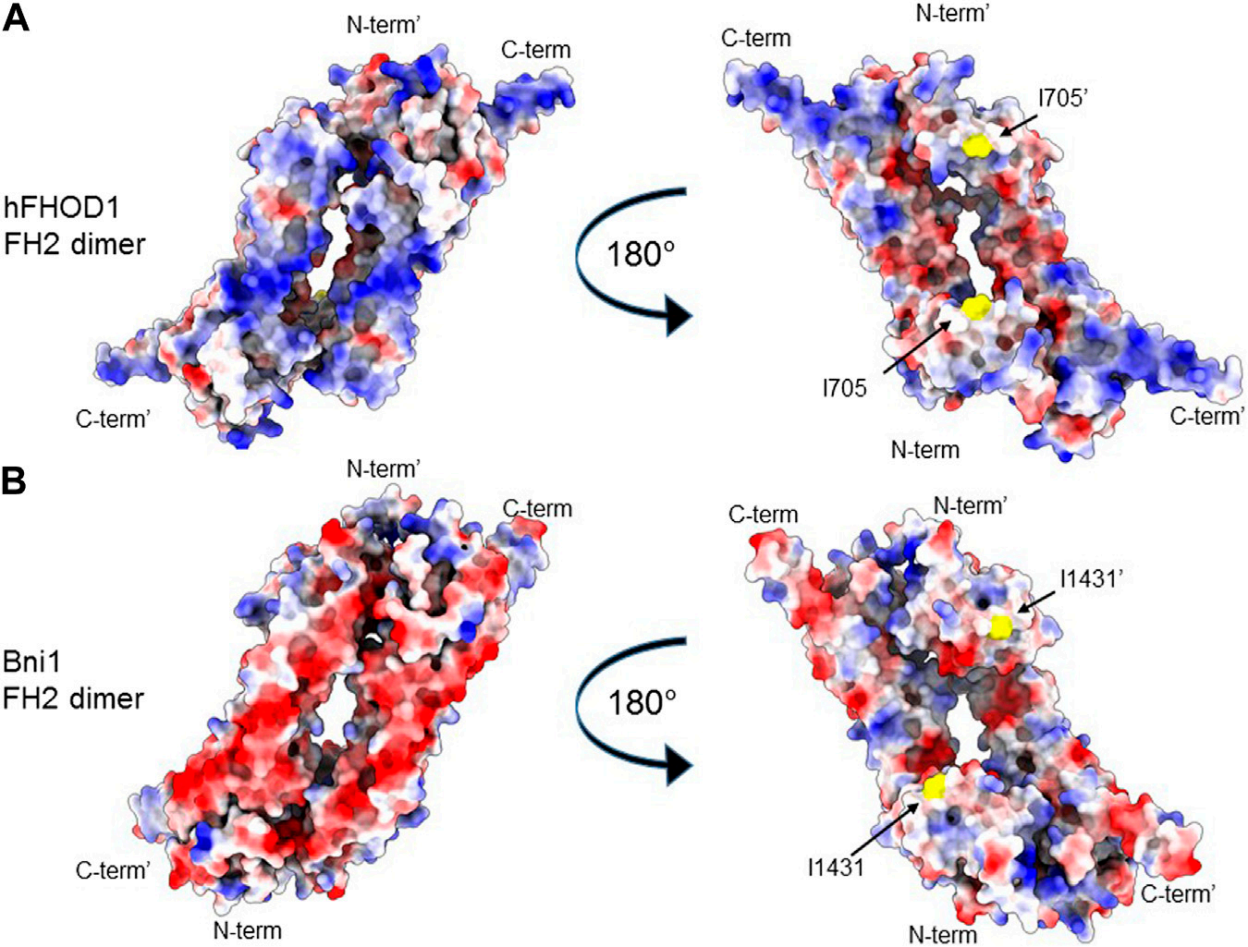
FH2 surface charge of a bundling (hFHOD1) and a non-bundling (Bni1) formin. **(A, B)** Alphafold2 model of the FH2 dimer of hFHOD1 **(A)** based on the experimental FH2 dimer of yeast Bni1 (PDB: 1ux4). **(B)** Identical views of the back and front surface of the dimer are depicted. Surface representation, coloring indicating Coulomb potential (blue, positive potential; red, negative potential). Critical residue for actin polymerization, I705 in *FHOD3* and the homologous I1431 in Bni1, in yellow. Note the characteristically positively charged surface of hFHOD1’s FH2 domain on the side opposite that containing residue I705 involved in barbed end binding and actin polymerization.

The mechanistic basis for FHODs’ actin bundling activity is not resolved. Residues in the FH2 domain critical for binding the actin barbed end, e.g., I705 in human FHOD1, are not necessary for FHODs’ bundling activity (Patel et al., 2018; Antoku et al., 2019). This indicates that barbed-end binding uses a different binding interface compared to that used for bundling (Figure 1B). One possibility is that basic residues in the FH2 domain may facilitate binding to the acidic actin filament surface (Harris et al., 2006). In fact, there is a correlation between the high isoelectric point of formins’ FH2 domains and their ability to bundle actin filaments (Harris et al., 2006). For example, FHODs have the highest isoelectric point among formins and robustly bundle actin filaments. Examination of the surface charge on a structural model of FHOD1’s FH2 reveals basic patches on the flip side of the FH2 toroid from that involved in barbed end binding, which does not contain extensive basic patches (Figure 2A). For comparison, basic patches are not seen on either side of the FH2 of the yeast formin Bni1 (Figure 2B), which does not bundle actin filaments (Moseley and Goode, 2005).

Whereas the dimeric toroid structure of the FH2 domain is essential for barbed end binding and actin polymerization (see above), whether it is necessary for actin bundling by FHODs or other actin bundling formins has not yet been tested. Harris et al. noted that there was rapid exchange of subunits in the dimeric FH2 of mDia2 and FRL1, two actin bundling formins, whereas subunits did not exchange in mDia1, a non-bundling formin (Harris et al., 2006). Thus, it is possible that actin bundling by FHODs involves a distinct oligomeric state from that involved in actin polymerization.

The unique IDR of FHODs plays a critical role in regulating the actin bundling activity of the FH2 domain and may also contribute binding energy to the bundling. The IDR binds weakly to actin filaments *in vitro* and is necessary for active forms of FHOD1 to localize to actin filaments in cells (Takeya and Sumimoto, 2003; Schonichen et al., 2013). Phosphorylation of residues in this region by ERK1/2 strongly decreases the actin bundling activity *in vitro* and in cells (Antoku et al., 2019). Given that the FH2 domain is sufficient for bundling, phosphorylation of the IDR may impact bundling of the FH2 domain through conformational change or intramolecular interaction.

#### FHOD autoregulation and post-translational modification

Similar to DRFs, actin activities of FHODs are controlled by autoregulatory interaction between their DID and DAD domains. However, unlike DRFs, Rho-dependent protein kinase ROCKI/II, rather than Rho/Cdc42 GTPase binding, regulates this interaction (Takeya et al., 2008; Zhou et al., 2017). ROCKI/II phosphorylation of conserved residues in the DAD of widely expressed human isoforms of FHOD1 (hFHOD1-2, residues S1131/S1137/T114) or *FHOD3* (hFHOD3-1, residues S1412/T1416), results in the dissolution of the DID-DAD intramolecular interaction activating FHOD1/3’s actin polymerization and bundling activities. ROCKI/II also phosphorylate the DAD of mDia2 (Staus et al., 2011), suggesting that this mode of regulation may be more common for formins than initially appreciated.

ERK1/2 kinases regulate the bundling activity of FHODs by phosphorylating conserved residues in the IDR between the DID and FH1 domains (Antoku et al., 2019). ERK1/2 phosphorylation of a single Ser residue (S498) in human FHOD1-2 or two (S497/S523) in mouse cardiac *FHOD3*-1 dramatically reduces their bundling activity *in vitro* and *in vivo*. ERK1/2 phosphorylation of *FHOD3* is elevated in a laminopathy mouse model (*Lmna* ^*H222P/H222P*^) causing cardiomyopathy and this disrupts the positioning and shape of the nucleus. Consistent with this, expression of a non-phosphorylatable version of *FHOD3* rescues defective nuclear positioning in fibroblasts expressing lamin A H222P (Antoku et al., 2019).

Src kinase phosphorylates hFHOD1-2 on Y99, which is not conserved in *FHOD3* (Iskratsch et al., 2013). Preventing Src phosphorylation of Y99 reduces ROCKI/II-mediated phosphorylation of DAD during cell attachment. This suggests that Y99 phosphorylation of FHOD1 is a prerequisite for ROCKI/II phosphorylation of DAD and hence FHOD1 activation during attachment.

Degradation of cardiac *FHOD3* is regulated by casein kinase 2 (CK2). CK2 phosphorylates two residues (T1476 and T1478) in the eight residue insert of the cardiac isoform hFHOD3-3 (Iskratsch et al., 2010). These residues are located near the carboxy-terminus of the FH2 domain. Phosphorylation of *FHOD3* by CK2 prevents its interaction with p62/SQSTM1 (sequestosome) and hence *FHOD3* degradation by autophagosomes.

Lastly, Aurora kinase B phosphorylates FHOD1 in the region between DID and FH1 (Floyd et al., 2013). The effect of this phosphorylation on the biochemical activities of FHOD1 is unknown, but in the cell it seems to reduce cortical F-actin formation.

#### FHOD tethering to the nucleus and nuclear functions

Among formins, FHODs are unique in their tethering to the nuclear membrane. They do so through interaction with the outer nuclear membrane localized giant nesprins, nesprin-1G/2G. These nesprins are integral membrane proteins composed of a large cytoplasmic domain containing many SRs, paired calponin homology (CH) domains, a single transmembrane domain, and a Klarsicht/ANC-1/Syne homology (KASH) domain that extends into the lumen between the outer and inner nuclear membranes. There, the KASH domain binds to inner nuclear membrane SUN proteins to form the linker of nucleoskeleton and cytoskeleton (LINC) complex that spans both nuclear membranes (Crisp et al., 2006; Starr and Fridolfsson, 2010; Sosa et al., 2012; Chang et al., 2015). The LINC complex is a central player in nuclear positioning and mechanotransduction (Gundersen and Worman, 2013).

**FIGURE 3:**
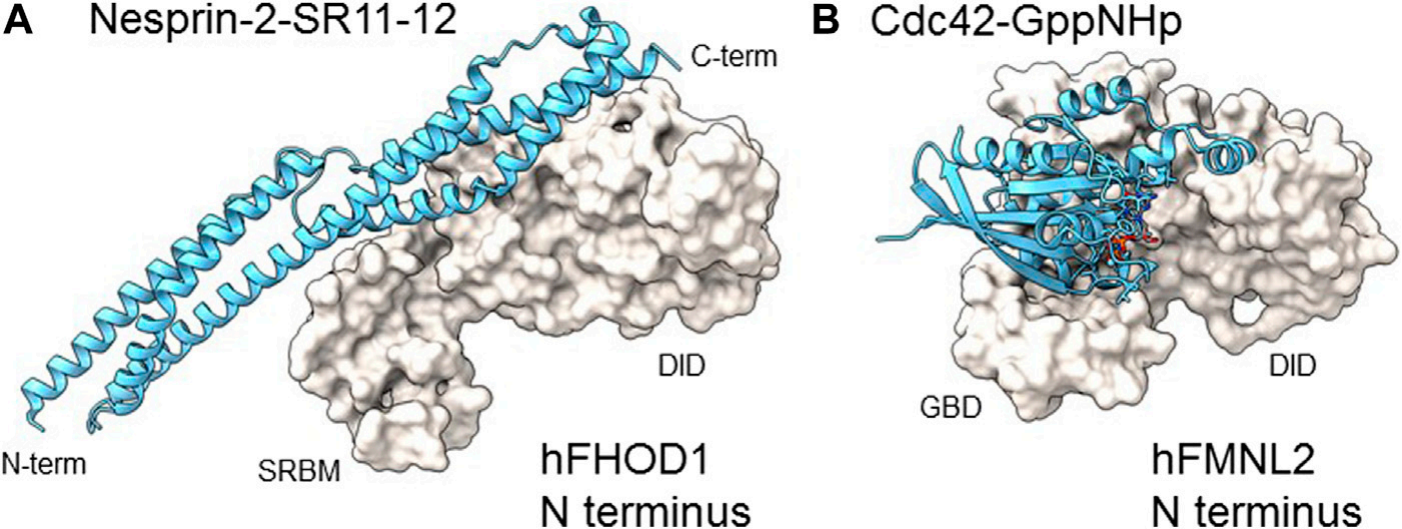
Comparison of N-terminal domain complexes of hFHOD1 and hFMNL2. **(A)**. Structure of the SRBM-DID module of hFHOD1 complexed to SR11 and 12 of nesprin-2G (PDB: 6xf1) compared to **(B)** the GBD-DID module of hFMNL2 complexed to GppNHp-bound Cdc42 (PDB: 4yc7). Domain abbreviations as in the legend of Figure 1A. The structure of the hFHOD1: nesprin-2G SR11-12 complex was modified from (Lim et al., 2021); that of hFMNL2-Cdc42-GppNHp was from (Kuhn et al., 2015).

FHOD1 was identified as a nesprin-2G interacting partner by a yeast two-hybrid screen and shown to bind directly to specific SRs in nesprin-2G (Kutscheidt et al., 2014). The recently reported 3D structure of the complex between nesprin-2G’s SR11-12 and FHOD1’s amino terminal regulatory domain revealed an interacting interface clearly distinct from that of Rho/Cdc42 GTPase binding formins, such as mDia1 or FMNL2 (Figures 3A, B) (Lim et al., 2021). The amino terminal region of FHOD1 that binds to SR11-12 of nesprin-2G was previously thought to bind to Rac GTPase and was called the GBD (GTPase binding domain) by analogy to other formins (Bechtold et al., 2014). However, a number of studies failed to find high affinity Rac binding (Schulte et al., 2008; Lim et al., 2021). The recent crystal structure resolved this issue by clearly showing high affinity interaction of this domain with nesprin SRs supporting its renaming as SRBM (Lim et al., 2021).

The identification of interacting residues in the FHOD1-nesprin-2G complex revealed a motif (DxWLD[IVLA]xE) in SR-11-12 that is 100% conserved in SR17-18 of nesprin-1G but not in other SRs of nesprins or other SR proteins (Lim et al., 2021). This motif was shown to be important for FHOD1 binding to both nesprins. The structure of the complex also allowed the identification of binding site point mutants for both FHOD1 and nesprin-2G, which provide powerful means to assess whether the interaction of FHOD1 with nesprin-1G/2G and the nucleus is required for their cellular functions. Additional data indicate that *FHOD3* interacts with nesprin-1G/2G by a similar mechanism (Antoku et al., 2019).

**FIGURE 4:**
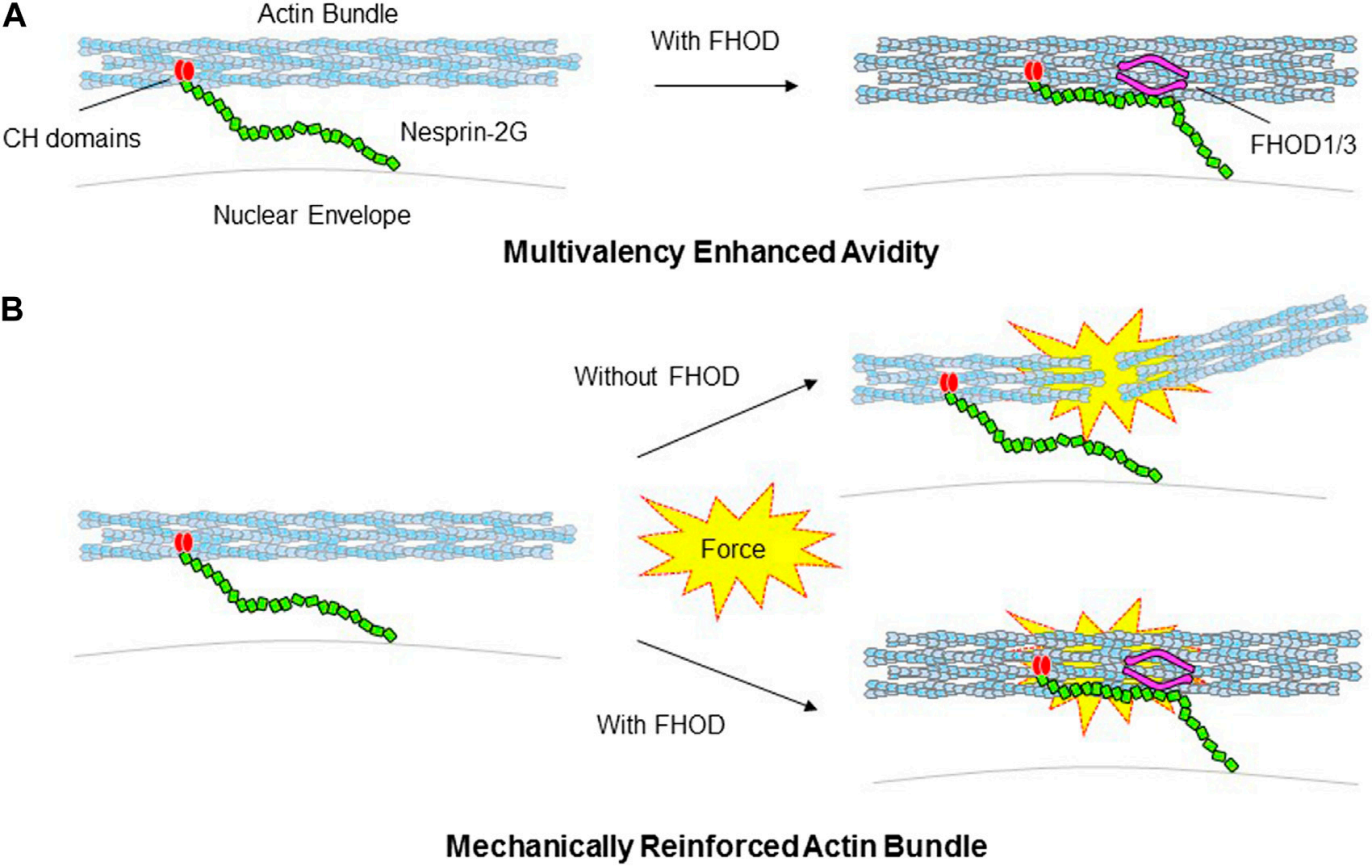
Models for how FHOD1/3 nuclear tethering influences nesprin’s interaction with actin bundles. **(A)**. Schematic depicting FHOD1/3 enhanced avidity of nesprin-2G for actin filaments due to multi-point attachment *via* nesprin-2G CH domains and FHOD1/3. **(B)**. Schematic depicting FHOD1/3 enhanced mechanical resistance of actin bundles engaged by nesprin-2G due to FHOD1 reinforcement of actin bundling.

Tethering of FHOD1/3 to the LINC complex enhances nuclear force transduction in two ways. It provides a second F-actin binding site adjacent to that provided by the paired CH domains within nesprin-1G/2G (Figure 4A). This branched connection is expected to enhance the avidity of the LINC-FHOD1/3 complex for actin filaments. It also provides enhanced mechanical resistance of the engaged F-actin cables through FHODs’ strong actin bundling activity (Figure 4B). This latter activity may be especially important for nuclear movement where the large forces required may otherwise disrupt the integrity of actin bundles.

One of the best-characterized FHOD1 pathways is that controlling nuclear positioning in fibroblasts polarizing for cell migration. In this pathway, serum, or the serum factor lysophosphatidic acid, activates Cdc42 to trigger actin retrograde flow to move the nucleus and microtubule cortical interactions to maintain the centrosome at the cell centroid (Palazzo et al., 2001; Gomes et al., 2005; Schmoranzer et al., 2009). Actin dependent movement of the nucleus requires the LINC complex components, nesprin-2G and SUN2, the inner nuclear membrane protein emerin and lamin A/C (Luxton et al., 2010; Folker et al., 2011; Chang et al., 2013). FHOD1 was identified as the first cytoplasmic factor (other than actin) that is recruited to the LINC complex during nuclear movement and is required for coupling actin cables to nesprin-2G (Kutscheidt et al., 2014). Point mutants in either FHOD1 or nesprin-2G that disrupt their interaction, prevent nuclear movement, showing that FHOD1 must be tethered to the nucleus for proper function (Lim et al., 2021). A similar FHOD1 and LINC complex-dependent pathway functions to recenter the nucleus in fibroblasts after displacement by centrifugal force (Zhu et al., 2017). In cardiomyocytes, *FHOD3* likely plays the same role in nuclear positioning as FHOD1 in fibroblasts (Antoku et al., 2019).

FHOD formins have also emerged as key focal points for the regulation of LINC complex mechanotransduction. ERK1/2 phosphorylate both FHODs and this downregulates their actin bundling, but not their actin polymerization activity, *in vitro* (Antoku et al., 2019). A single site is phosphorylated in human FHOD1 and two in mouse cardiac isoform *FHOD3* (see above). These sites are within the IDR of the FHODs providing further evidence that this region is important for bundling. In fibroblasts, ERK1/2 phosphorylation of FHOD1 impairs its ability to bundle actin filaments and restricts actin-dependent nuclear movement (Antoku et al., 2019). Thus, phosphorylation of FHODs acts as an on-off switch for mechanical engagement of the LINC complex with the actin cytoskeleton.

FHOD1 expression is upregulated in human myoblasts expressing pathogenic mutations in *LMNA* (encoding lamin A/C) or *Syne-1* (encoding nesprin-1) providing further evidence for the co-dependency of FHODs and the LINC complex (Schwartz et al., 2017). However, *FHOD3* expression is unchanged in hearts of mice expressing lamin A H222P, a model of dilated cardiomyopathy, so upregulation of FHODs by LINC complex disruption may not be typical of all cell types (Antoku et al., 2019).

#### FHOD functions beyond the nucleus

FHODs have been implicated in functions at sites other than the nuclear surface. These include the formation of substrate adhesions and actin protrusions during cell migration (Koka et al., 2003; Gardberg et al., 2013; Iskratsch et al., 2013; Lammel et al., 2014; Paul et al., 2015; Monzo et al., 2016). Another is in the assembly of striated muscle sarcomeres (Taniguchi et al., 2009; Kan et al., 2012; Mi-Mi et al., 2012; Fujimoto et al., 2016; Shwartz et al., 2016; Fenix et al., 2018). With the possible exception of *FHOD3*’s function in sarcomeres, none of these studies has described a tethering mechanism to localize FHODs, so how directly FHODs affect these processes is unknown.

FHODs have a role in fibroblast, cancer and immune cell migration in 1D, 2D and 3D environments (Koka et al., 2003; Gardberg et al., 2013; Iskratsch et al., 2013; Lammel et al., 2014; Paul et al., 2015; Monzo et al., 2016; Heuser et al., 2020). FHODs may facilitate migration by contributing to nuclear positioning through the LINC complex (Luxton et al., 2010; Chang et al., 2015). Other studies support functions of FHODs at sites distal to the nucleus. FHOD1 is implicated in the formation of actin filaments to cluster integrin receptors before their maturation into focal adhesions (Iskratsch et al., 2013). In 3D migration of carcinoma cells, *FHOD3* is required for actin microspike formation at the cell front after integrin recycling (Paul et al., 2015). In both these cases (and typical of many studies of FHODs), the idea that FHODs are acting directly on these structures is supported by localization of overexpressed FHOD proteins and by consideration of FHODs ability to polymerize actin. Yet, FHODs’ relatively weak actin polymerization activity raises the question of how it contributes to these processes, which usually require rapid actin polymerization. Additionally, overexpressed and activated FHODs decorate virtually all actin filaments in cells so the localizations based on this approach are not unequivocal evidence for localized action of FHODs. Given recent studies showing that the LINC complex can influence cell-substratum adhesions through its connections to the cytoskeleton (Woychek and Jones, 2019; Carley et al., 2021), it is worth considering that FHODs may influence distant actin arrays by exerting force on them through their tethering to the LINC complex.

Mice lacking FHOD1 do not have an observable phenotype, perhaps reflecting compensation by *FHOD3* or other formins (Sanematsu et al., 2019). In contrast, mice lacking *FHOD3* die embryonically due to improper heart formation resulting from defective myofibrillogenesis (Taniguchi et al., 2009; Kan et al., 2012). Consistent with this, conditional knockout of *FHOD3* or expression of the actin polymerization defective mutant I1127A only affect cardiac sarcomeres embryonically (Fujimoto et al., 2016; Ushijima et al., 2018). Disruption of the single FHOD family homologues in *C. elegans* or *D. melanogaster* also leads to cardiac developmental defects showing this is an evolutionary conserved function (Mi-Mi et al., 2012; Shwartz et al., 2016; Sundaramurthy et al., 2020).

A role for *FHOD3* in sarcomerogenesis has been observed by replating induced pluripotent stem cell-derived cardiomyocytes (iPSC-CM) (Fenix et al., 2018). IPSC-CMs lacking the cardiac isoform of *FHOD3* do not reform sarcomeres after replating. Similar to *FHOD3*, nesprin-1 and 2 are also needed for normal cardiac formation and seem to play a role in cardiac morphogenesis (Banerjee et al., 2014). During *D. melanogaster* muscle development sarcomeres initially form near nuclei (Auld and Folker, 2016). One way for the nucleus and nuclear FHOD to participate in sarcomerogenesis would be to enhance the initial formation of sarcomeric F-actin through its actin polymerization and bundling activities. Consistent with this idea, bundles of actin filaments accumulate at the periphery of the nucleus during sarcomerogenesis in iPSC-CM (Chopra et al., 2018; Fenix et al., 2018).

A mechanism for tethering *FHOD3* to cardiac sarcomeres has been established. Cardiac specific *FHOD3* localizes to the center of the M band in sarcomeres and this does not require its barbed end binding (Matsuyama et al., 2018). Instead, this localization is due to its direct interaction with myosin-binding C-protein (MyBCP), which also localizes to this site (Matsuyama et al., 2018). MyBCP acts as a sarcomeric tether for *FHOD3*, as mice null for MyBCP lack *FHOD3* in their cardiac sarcomeres and develop hypertrophic cardiomyopathy. The sarcomeres in MyBCP null mice appear to form normally indicating that localization of *FHOD3* to sarcomeres is not required for sarcomere formation (Matsuyama et al., 2018). Perhaps *FHOD3*’s tethering to MyBCP contributes in some other way to sarcomere function.

In addition to cell migration and sarcomere formation, there are other reported functions of FHODs. These include sensing environment stiffness in neonatal cardiomyocytes (Pandey et al., 2018), closure of the neural plate (Sulistomo et al., 2019), regulation of dendrite spine morphology in a subset of pyramidal neurons in the cerebral cortex (Sulistomo et al., 2021), contractile ring constriction during cytokinesis in (Terry et al., 2018), SRF transcriptional activation (Westendorf, 2001; Madrid et al., 2005), and intracellular pathogen invasion (Alvarez and Agaisse, 2013; Truong et al., 2013). Whether these involve FHOD tethering to the nucleus or to other sites still needs to be tested.

#### FHOD1/3 functions in disease

Mutations in *FHOD3* are strongly linked to hypertrophic cardiomyopathy (Ochoa et al., 2018; Huang et al., 2020; Ochoa et al., 2020) and are also associated with dilated cardiomyopathy (Arimura et al., 2013). The genetic basis for mutations causing hypertrophic cardiomyopathy is particularly strong. The mutations are spread throughout the *FHOD3* gene but occur recurrently in at least two sites (S527del and Y528C) from unrelated families. These sites are in one of the exons inserted in the cardiac specific isoform. A second cluster of mutations resides adjacent to these recurrent mutations and “hits” Arg residues positioned every 3-4 residues. These variants map to the region of *FHOD3* required for its bundling activity, but they have not yet been tested for effects on *FHOD3* activities or whether they affect sarcomere formation. The variant *FHOD3* associated with dilated cardiomyopathy was in the FH2 domain and appeared to disrupt *FHOD3*’s ability to stimulate SRF-dependent transcription, an activity dependent on formin stimulation of actin polymerization (Arimura et al., 2013). The association of *FHOD3* with cardiac disease is consistent with mouse knockout studies showing *FHOD3* plays a role in normal cardiac development and homeostasis (Kan et al., 2012; Ushijima et al., 2018).

*FHOD1* expression is upregulated in many cancers including glioma, melanoma, squamous cell, gastric, and breast cancers (Jurmeister et al., 2012; Gardberg et al., 2013; Peippo et al., 2017; Heuser et al., 2020; Jiang et al., 2021). Upregulated expression correlates with poor survival (Heuser et al., 2020; Jiang et al., 2021). Reduction of FHOD1 expression in these cancer cells decreases cell proliferation, colony formation *in vitro*, cell migration, and invasiveness (Jurmeister et al., 2012; Gardberg et al., 2013; Peippo et al., 2017; Jiang et al., 2021). In breast cancer cells, FHOD1 is a target of downregulation by miRNA-200c (Jurmeister et al., 2012). Reducing the expression of FHOD1 in the breast cancer cells decreases the activity of SRF through reduced actin filament formation. Experimentally reducing FHOD1 expression in squamous cancer cells also reduces filamentous actin (Gardberg et al., 2013). Thus, it is likely that one of the key mechanisms for how upregulated FHOD1 expression promotes cancer progression is through increased SRF activity. In addition, FHOD1 positively regulates the formation of clustered “rosettes” of invadopodia (Gulvady et al., 2019). This most likely contributes to the invasiveness of cancer cells (Gardberg et al., 2013; Peippo et al., 2017) and promotes changes in the secretion of extracellular membrane proteins (Jiang et al., 2021). In contrast to FHOD1, *FHOD3* upregulation has not been reported in cancer cells. However, *FHOD3* is reported to play a positive role in glioma and ovarian cancer cells by promoting cell migration (Paul et al., 2015; Monzo et al., 2016).

## Perspectives

FHODs are a separate formin family based on their sequence differences with other formins and their activities, which include strong actin filament bundling, weak actin polymerization and unique association with actin bundles, nesprins and MyBP-C. We have discussed structural, biochemical and cell biological evidence supporting a clear function for both FHOD1/3 in tethering to the nucleus to mediate mechanical force transmission. For this function, FHODs act as critical components of the LINC complex connection to actin filaments, reminiscent of proteins such as talin that couple membrane bound integrins to the actin cytoskeleton. This now adds functional characteristics of FHODs to the features that distinguish them as a separate class of formins. Whether the LINC complex-associated function of FHODs is involved in additional processes, for example adhesion formation during cell migration or sarcomere formation, can now be tested given the availability of variants in FHODs and nesprin-1/2 that block their interaction. Whether there are distinct tethers that localize FHODs to other cellular locales needs further exploration, but MyBP-C is a strong candidate for a non-nuclear tether. Understanding how FHODs are tethered to sites of function will contribute further to understanding how mechanotransduction affects specific sites in cells both in normal physiology and in disease.
